# Plasma FABP2, IL-10, and LPS in Microscopic Colitis: An Exploratory Study of Their Biomarker Potential

**DOI:** 10.3390/medicina62071237

**Published:** 2026-06-26

**Authors:** Vytautas Kiudelis, Greta Gedgaudienė, Justina Veličkienė, Dalius Petrauskas, Jurgita Skiecevičienė, Juozas Kupčinskas, Gediminas Kiudelis, Laimas Virginijus Jonaitis

**Affiliations:** 1 Department of Gastroenterology, Lithuanian University of Health Sciences, Eiveniu St. 2, LT-50161 Kaunas, Lithuania; dalius.petrauskas@lsmu.lt (D.P.); juozas.kupcinskas@lsmu.lt (J.K.); gediminas.kiudelis@lsmu.lt (G.K.); 2 Institute for Digestive Research, Lithuanian University of Health Sciences, Eiveniu St. 4, LT-50161 Kaunas, Lithuania; greta.gedgaudiene@lsmu.lt (G.G.); justina.velickiene@lsmu.lt (J.V.); jurgita.skieceviciene@lsmu.lt (J.S.)

**Keywords:** microscopic colitis, collagenous colitis, lymphocytic colitis, ulcerative colitis, fatty acid-binding protein 2, interleukin-10, lipopolysaccharides, biomarker

## Abstract

*Background and Objectives*: Microscopic colitis (MC) encompasses two chronic inflammatory disorders of the large intestine: collagenous colitis (CC) and lymphocytic colitis (LC). Both conditions are characterised by chronic watery diarrhoea and substantially impaired quality of life. Diagnosis relies on colonoscopy with multiple biopsies, and no reliable non-invasive biomarker currently exists. This exploratory study aimed to investigate circulating fatty acid-binding protein 2 (FABP2), interleukin-10 (IL-10), and lipopolysaccharides (LPSs) as potential biomarkers for MC and to compare their profiles with those in ulcerative colitis (UC). *Materials and Methods*: Plasma samples were obtained from 45 patients with active CC, 16 patients with active LC, 52 healthy controls, 43 patients with active UC, and 43 patients with inactive UC. Concentrations of FABP2, IL-10, and LPS were measured by enzyme-linked immunosorbent assay (ELISA). *Results*: Plasma FABP2 concentrations differed significantly across groups (Kruskal–Wallis *p* = 0.008). CC patients exhibited the highest levels (median 1719.0 pg/mL, IQR 1364.0–2240.0) compared with active UC (median 1272.0 pg/mL, IQR 861.7–1727.5; *p* = 0.005) and inactive UC (median 1334.0 pg/mL, IQR 854.2–1702.0; *p* = 0.001), but did not differ significantly from controls (median 1364.5 pg/mL, IQR 982.6–2160.5; *p* = 0.076) or LC (median 1421.5 pg/mL, IQR 1207.0–2002.2; *p* = 0.171). IL-10 concentrations also differed across groups (Kruskal–Wallis *p* = 0.029 after removal of one extreme outlier in active UC). Active UC patients had significantly lower levels (median 2.6 pg/mL, IQR 1.4–4.6) than CC (median 4.0 pg/mL, IQR 3.0–7.2; *p* = 0.009) and controls (median 4.8 pg/mL, IQR 2.6–7.4; *p* = 0.005). LPS concentrations showed no overall differences across groups (Kruskal–Wallis *p* = 0.55), although CC patients had numerically higher levels (median 73.7 pg/mL, IQR 45.6–104.9) compared with controls (median 56.4 pg/mL, IQR 33.7–87.1; *p* = 0.124). No significant differences were observed between LC and other groups for any biomarker. *Conclusions*: In this exploratory study, plasma FABP2 and IL-10 showed limited diagnostic accuracy in differentiating CC from UC but failed to distinguish MC from healthy controls. LPS levels were not significantly different among study groups. None of the biomarkers reliably separated LC from other groups, possibly reflecting the small LC sample size. These preliminary findings suggest subtle differences in circulating biomarker profiles between CC and UC that warrant validation in larger cohorts.

## 1. Introduction

Microscopic colitis (MC) encompasses two main chronic inflammatory disorders of the large intestine: collagenous colitis (CC) and lymphocytic colitis (LC). Both subtypes are characterized by chronic watery diarrhea, frequently accompanied by nocturnal bowel movements, urgency, and, in some cases, fecal incontinence. These symptoms significantly impair patients’ quality of life [1,2,3]. A hallmark of MC is the absence of macroscopic mucosal damage; the colonic mucosa typically appears normal or near-normal on colonoscopy, in contrast to classic inflammatory bowel diseases (IBDs) such as ulcerative colitis (UC) and Crohn’s disease (CD) [1]. Consequently, diagnosis requires a high index of clinical suspicion, as confirmation relies on histopathological evaluation of biopsy specimens obtained from endoscopically normal-appearing mucosa [1]. Histologically, LC is defined by an increased intraepithelial lymphocyte count (≥20 per 100 epithelial cells) together with enhanced lamina propria cellularity, whereas CC is characterized by a thickened subepithelial collagen band (≥10 µm) and increased lamina propria cellularity [1]. Oral budesonide remains the treatment of choice for MC, being the only agent thoroughly evaluated in randomized clinical trials [4,5,6,7,8,9,10]. However, relapse occurs in more than 80% of patients after budesonide withdrawal, and approximately half of individuals experience frequently relapsing or chronically active disease that necessitates long-term therapy [5,9,11]. Non-invasive biomarkers routinely used in IBD, such as C-reactive protein (CRP) and faecal calprotectin, demonstrate limited or no diagnostic utility in MC [1]. Although certain faecal markers, including chromogranin A and eosinophil granule proteins, have shown elevation in some studies [12,13], no validated biomarker for MC is currently available.

Fatty acid-binding protein 2 (FABP2) is expressed in the epithelial layer of both the small and large intestine—primarily in enterocytes and, to a lesser extent, in colonocytes—and plays a key role in the intracellular buffering and transport of long-chain fatty acids [14]. Circulating FABP2 levels increase when the small intestinal mucosal barrier is disrupted, a finding observed in both chronic and acute conditions such as coeliac disease and necrotising enterocolitis, respectively [15,16]. The diagnostic utility of FABP2 in IBD has been investigated extensively, while early studies suggested promise in CD [17,18], subsequent research has demonstrated limited value as a diagnostic marker in both CD and UC. This is thought to result from chronic inflammation depleting FABP2-producing cells [19,20]. However, a recent study has proposed that FABP2 may contribute directly to CD pathophysiology by exacerbating dysbiosis and promoting inflammation when hijacked by Enterococcus faecalis [21].

Interleukin-10 (IL-10) is an anti-inflammatory cytokine that plays a key role in downregulating proinflammatory signals within the mucosal immune response [22]. Studies have shown that IL-10 levels are elevated in UC; however, concentrations are significantly higher in patients with inactive or mildly active UC and CD compared with those with moderately or severely active disease, possibly reflecting resolution of inflammation [23,24]. Furthermore, IL-10 concentrations have been shown to decrease in steroid-refractory patients and to rise again following rescue therapy [25]. One study demonstrated higher IL-10 levels in colonic tissue from both CC and LC patients and suggested that this may explain why inflammation in MC does not typically progress to ulceration and structural damage [26]. However, circulating IL-10 concentrations in patients with MC have not yet been studied.

Lipopolysaccharides (LPSs) are glycolipid endotoxins originating from Gram-negative gut bacteria [27]. Under normal circumstances, a healthy intestinal barrier prevents LPSs from entering the systemic circulation. However, when the integrity of the barrier is disrupted, LPSs may translocate into the bloodstream and trigger inflammation via Toll-like receptor 4 on immune cells. The presence of LPSs in systemic circulation is therefore considered a marker of impaired intestinal barrier function [28]. In CD, LPS levels have been found to be increased in active disease compared with controls and to correlate with disease activity [28,29]. Similar findings have been reported in UC [30]. However, another study found that LPS levels in IBD patients were comparable to those in controls, although there was evidence of prior LPS exposure [31]. No studies have yet evaluated circulating LPSs in MC, despite evidence suggesting that intestinal barrier disruption plays a role in MC pathogenesis [32]. Studies have demonstrated reduced transepithelial resistance in both CC and LC, decreased expression of tight junction proteins in LC, and increased uptake of *E. coli* K12 bacteria in CC that diminishes after treatment with budesonide [33,34]. Although limited, the evidence suggests that epithelial barrier dysfunction plays an important role in MC pathogenesis.

To date, no established biomarkers are available for MC. Standard laboratory tests routinely used in UC and CD, such as CRP and faecal calprotectin, show only mild or no elevation in MC and therefore have limited diagnostic value [1,35]. In light of this unmet need, we conducted an exploratory study to investigate whether assessing FABP2, IL-10, and LPS levels could help differentiate CC and LC from healthy controls and UC patients.

## 2. Materials and Methods

### 2.1. Patient Enrollment and Sample Collection

This cross-sectional cohort study was conducted between 2019 and 2022. Plasma samples were collected from patients with newly diagnosed active CC and LC at the Department of Gastroenterology, Hospital of Lithuanian University of Health Sciences Kauno Klinikos, Lithuania. Peripheral blood samples were obtained using 5 mL plasma separator tubes (Becton, Dickinson and Company, Franklin Lakes, NJ, USA) for subsequent plasma isolation. The diagnosis of CC and LC was established according to histopathological criteria based on international guidelines: CC was defined by a thickened subepithelial collagen layer (≥10 µm) with increased inflammatory cells in the lamina propria, while LC was characterised by an increased number of intraepithelial lymphocytes (≥20 per 100 epithelial cells) with increased lamina propria cellularity and no significant subepithelial collagen thickening [1]. Disease activity in MC patients was assessed using the Hjortswang criteria, with active disease defined as an average of ≥3 bowel movements per day or ≥1 watery bowel movement per day over a 7-day period [35]. Control participants were recruited from the colorectal cancer screening programme. Individuals were eligible if they reported no abdominal symptoms, normal bowel habits, no medication use, and had no pathological findings on colonoscopy. For comparative purposes, patients with UC were recruited from the IBD centre of the Department of Gastroenterology at the same institution. Active UC patients were enrolled during disease flares, whereas inactive UC patients were recruited during routine follow-up visits while in clinical and endoscopic remission. Disease activity in UC was determined using the Mayo score and Mayo endoscopic score. All UC patients had a previously confirmed diagnosis based on clinical, endoscopic, and histological criteria in accordance with international guidelines [36]. Exclusion criteria for all groups included signs of active infection, prior colorectal surgery and antibiotic use within the last 3 months. All participants provided written informed consent, and the study was approved by the Kaunas Regional Biomedical Research Ethics Committee (No. P3-BE-2-31/2018).

### 2.2. Analyses of FABP2, IL-10 and LPSs by ELISA

Circulating levels of the selected protein biomarkers were quantified using sandwich ELISA technology. The following commercially available kits were employed: (i) Human FABP2/I-FABP Quantikine ELISA Kit (catalogue no. DFBP20; R&D Systems, Minneapolis, MN, USA) for FABP2, (ii) Human IL-10 Quantikine ELISA Kit (catalogue no. D1000B; R&D Systems, Minneapolis, MN, USA) for IL-10, and (iii) Human Lipopolysaccharides ELISA Kit (catalogue no. CSB-E09945h; Cusabio, Wuhan, China) for LPSs. All samples were analysed in duplicate to ensure reproducibility. Optical density readings were measured using a Tecan Sunrise microplate absorbance reader (Tecan Group Ltd., Männedorf, Switzerland) at 450 nm, with 570 nm as the reference wavelength. Sample processing, incubation steps, washing procedures, and initial data handling were performed in strict accordance with each manufacturer’s protocol.

Standard curve fitting and concentration calculations were performed using a four-parameter logistic (4PL) regression model to generate the standard curves and interpolate unknown sample concentrations. This non-linear regression approach is the preferred method for most ELISA assays, as it accurately models the characteristic sigmoid shape of the dose–response curve across the full dynamic range, accounting for lower and upper asymptotes, the inflection point (EC50), and the Hill slope. Specifically, standard concentrations and corresponding absorbance values (after blank subtraction) were entered into the free online ELISA Calculator available at https://biotoolskit.com/protein-tools/elisa-calculator (accessed on 14 January 2022). The 4PL fitting module was selected, and sample absorbances were input (with appropriate dilution factors applied where relevant). The tool automatically computed fitted parameters, provided goodness-of-fit metrics (including R^2^), and calculated final analyte concentrations for each sample. Values falling outside the validated range of the standard curve were flagged as out-of-range and, when necessary, prompted re-analysis at adjusted dilutions. Results exceeding the manufacturer-specified cut-off values were classified as positive. This standardised computational workflow ensured high precision, traceability, and consistency in biomarker quantification across the exploratory cohort.

### 2.3. Statistics

Statistical analysis was performed using R (v4.3). Biomarker concentrations were assessed for normality (Shapiro–Wilk test) and homogeneity of variances. Due to frequent non-normality, group comparisons across the five independent groups (CC, LC, active UC, inactive UC, controls) were conducted using the Kruskal–Wallis test (omnibus) as the primary analysis. Post hoc pairwise comparisons (Mann–Whitney U tests) were performed with Bonferroni correction for multiplicity when the omnibus test was significant (*p* < 0.05) or for pre-specified contrasts. Results are reported as medians with interquartile ranges (IQRs) or means with 95% confidence intervals where appropriate. Receiver operating characteristic (ROC) curves and area under the curve (AUC) were generated for diagnostic performance. *p*-values < 0.05 were considered statistically significant.

## 3. Results

### 3.1. Baseline Characteristics

The baseline characteristics of the study population are summarized in Table 1. No significant differences in age were observed between most groups. Mean ages were comparable across CC (58 ± 13.4 years), LC (55 ± 16.1 years), controls (59 ± 4.9 years), and active UC (58 ± 8.8 years; all pairwise *p* > 0.15 by *t*-test). Inactive UC patients were slightly younger (54 ± 12.4 years), with a statistically significant difference only versus controls (*p* = 0.008). Significant differences existed in sex distribution (overall χ^2^ *p* = 0.004), driven by the strong female predominance characteristic of microscopic colitis. CC patients were 84% female (significantly higher than controls at 52%, *p* = 0.001, and inactive UC at 53%, *p* = 0.004).

### 3.2. FABP2 in Patients with MC and UC

**Figure 1:**
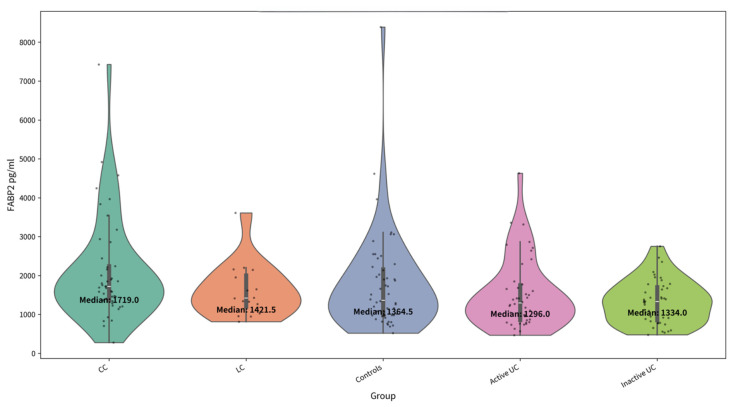
Plasma concentrations of FABP2. CC patients exhibited significantly higher levels of FABP2 compared to active UC (*p* = 0.005) and inactive UC (*p* = 0.001). FABP2: fatty acid-binding protein 2, UC: ulcerative colitis, CC: collagenous colitis, LC: lymphocytic colitis.

Plasma concentrations of FABP2 differed significantly across the five groups (Kruskal–Wallis *p* = 0.008). CC patients exhibited the highest levels (median 1719.0 pg/mL, IQR 1364.0–2240.0) compared to active UC (median 1272.0 pg/mL, IQR 861.7–1727.5; *p* = 0.005) and inactive UC (median 1334.0 pg/mL, IQR 854.2–1702.0; *p* = 0.001). No significant differences were observed between CC and controls (median 1364.5 pg/mL, IQR 982.6–2160.5; *p* = 0.076) or LC (median 1421.5 pg/mL, IQR 1207.0–2002.2; *p* = 0.171). FABP2 levels were comparable between active and inactive UC (Figure 1).

### 3.3. IL-10 in Patients with MC and UC

**Figure 2:**
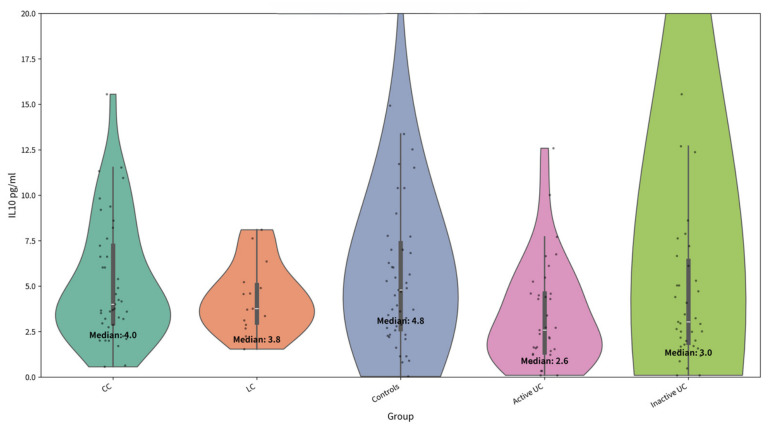
Violin plot of plasma concentrations of IL-10. Active UC patients had significantly lower plasma IL-10 compared to CC (*p* = 0.009) and controls (*p* = 0.005). IL-10: interleukin-10, UC: ulcerative colitis, CC: collagenous colitis, LC: lymphocytic colitis.

IL-10 concentrations showed overall differences across groups (Kruskal–Wallis *p* = 0.029 after removal of one extreme outlier in active UC). Active UC patients had significantly lower plasma IL-10 (median 2.6 pg/mL, IQR 1.4–4.6) compared to CC (median 4.0 pg/mL, IQR 3.0–7.2; *p* = 0.009) and controls (median 4.8 pg/mL, IQR 2.6–7.4; *p* = 0.005). Levels in LC (median 3.8 pg/mL, IQR 3.0–5.1) and inactive UC (median 3.0 pg/mL, IQR 1.9–6.4) were intermediate (Figure 2).

### 3.4. LPSs in Patients with MC and UC

**Figure 3:**
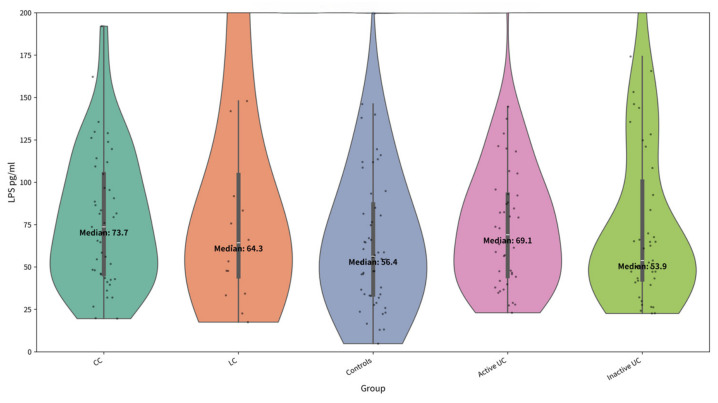
Plasma concentrations of LPSs. No significant overall differences in LPS concentrations were detected across groups. LPSs: lipopolysaccharides, UC: ulcerative colitis, CC: collagenous colitis, LC: lymphocytic colitis.

No significant overall differences in LPS concentrations were detected across groups (Kruskal–Wallis *p* = 0.55). CC patients showed numerically higher levels (median 73.7 pg/mL, IQR 45.6–104.9) compared to controls (median 56.4 pg/mL, IQR 33.7–87.1; *p* = 0.124), while active UC (median 65.2 pg/mL, IQR 43.0–92.6) and inactive UC (median 53.9 pg/mL, IQR 42.6–100.5) were similar to controls and LC (median 64.3 pg/mL, IQR 44.3–104.4) (Figure 3).

### 3.5. Diagnostic Accuracy of FABP2, IL-10 and LPSs

**Figure 4:**
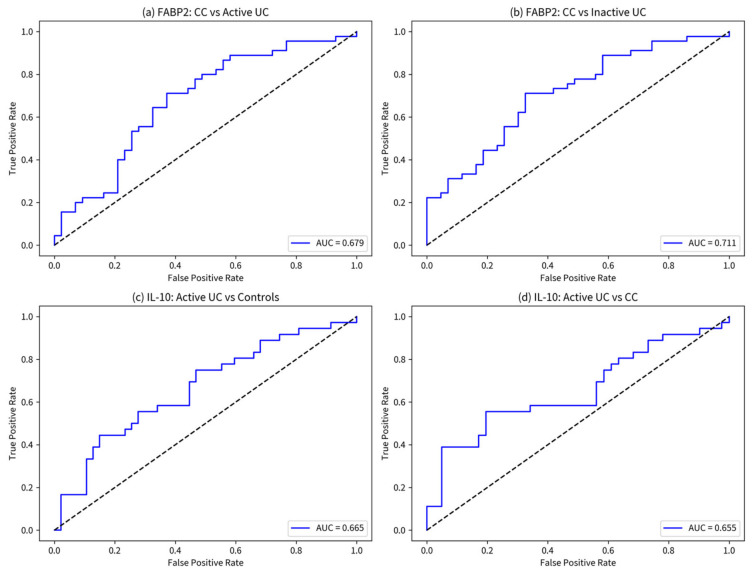
Diagnostic accuracy of FABP2 and IL-10: (**a**) ROC curve of FABP2 between CC and active UC; (**b**) ROC curve of FABP2 between CC and inactive UC; (**c**) ROC curve of IL-10 between active UC and controls; (**d**) ROC curve of IL-10 between active UC and CC. The diagonal dashed lines represent chance-level performance. FABP2: fatty acid-binding protein 2, IL-10: interleukin-10, UC: ulcerative colitis, CC: collagenous colitis, ROC: receiver operating characteristic, AUC: area under the curve.

ROC curve analyses were performed on the full dataset to evaluate the discriminatory performance of FABP2 and IL-10 for the statistically significant group comparisons (Figure 4). Plasma FABP2 demonstrated modest discriminatory ability in distinguishing CC from UC. For CC versus active UC, the AUC was 0.68 (95% CI approximately 0.56–0.80), with an optimal cutoff around 1440–1500 pg/mL yielding approximately 65–70% sensitivity and specificity. Similarly, for CC versus inactive UC, the AUC was approximately 0.71, with comparable performance metrics at a cutoff near 1450 pg/mL. For IL-10, ROC analysis highlighted its exploratory evidence of potential utility in differentiating active UC from both controls and CC. The AUC for active UC versus controls was approximately 0.67, with a cutoff near 2.7–3.0 pg/mL providing reasonable sensitivity (around 70%) but modest specificity. A similar AUC (~0.66) was observed for active UC versus CC.

## 4. Discussion

Because no established biomarkers exist for MC, representing an important unmet need in clinical practice [37], we describe for the first time the potential of FABP2, IL-10, and LPS as non-invasive biomarkers for MC. FABP2 has not previously been investigated as a biomarker in MC. In UC, it has not been found to increase consistently during flares, thereby limiting its diagnostic value. This observation is explained by the fact that FABP2 is expressed to a significantly greater extent in small intestinal epithelial cells than in colonic epithelial cells [14,38,39]. Indeed, Logan et al. reported a mean plasma FABP2 concentration of 1309 pg/mL in UC patients, which was not significantly elevated and could not reliably differentiate UC from controls, although it did distinguish patients with coeliac disease and newly diagnosed CD [18]. These findings align with our results, in which FABP2 also failed to differentiate UC from controls. Another study demonstrated elevated FABP2 levels in severe UC with backwash ileitis, suggesting that small intestinal involvement or extensive colonic damage is required for a rise in circulating FABP2 [40]. Interestingly, our study revealed significantly higher FABP2 levels in CC compared with both active and inactive UC, despite CC traditionally being regarded as a disease confined to the colon [41]. One possible explanation is the presence of undiagnosed coeliac disease within this cohort, given the well-documented association between MC and coeliac disease [42]. Alternatively, the elevated FABP2 concentrations may reflect subtle, subclinical small intestinal inflammation associated with MC—a hypothesis that warrants further investigation. It should also be noted that FABP2 did not significantly differentiate CC or LC from healthy controls in our study. These observations underscore the need for cautious interpretation and emphasise the importance of validating the findings in larger, independent cohorts, particularly given the small number of LC patients included.

IL-10 is an anti-inflammatory cytokine produced by immune cells, including regulatory T cells, B cells, dendritic cells, and macrophages. It plays a central role in controlling excess inflammation, and its deficiency has been shown to cause spontaneous development of early IBD [43]. Given this background, IL-10 would not be expected to behave as a typical biomarker that directly correlates with active inflammation. Indeed, Mitsuyama et al. demonstrated that IL-10 concentrations rise significantly during the resolution phase of UC and CD before returning to normal levels thereafter [23]. This may explain the lack of elevated IL-10 values in our inactive UC cohort, as many of these patients were in sustained remission rather than in the immediate post-flare resolution phase, during which IL-10 concentrations are expected to peak. Another study reported that IL-10 concentrations are higher in patients with UC and CD in remission than in those with active disease, and that levels are also higher in mildly active disease than in moderate or severe disease activity [24]. These observations are consistent with our findings, in which IL-10 concentrations in active UC were significantly lower than in controls and CC patients (who had concentrations similar to those in controls). This difference may be attributable to the moderate-to-severe disease activity in our active UC cohort, reflected by a mean Mayo score of 7.8 and a mean Mayo endoscopic score of 2. Carrasco et al. reported increased IL-10 levels in colonic tissue from both CC and LC patients, suggesting that enhanced local IL-10 production—together with higher numbers of regulatory T cells and double-negative T cells—may contribute to the absence of strong proinflammatory cytokine responses and the lack of progression to structural damage typically observed in MC [26]. In contrast, our study did not demonstrate a corresponding increase in circulating IL-10 levels in plasma from CC or LC patients. This discrepancy highlights the importance of distinguishing between local mucosal immune responses and systemic inflammatory profiles. The absence of elevated plasma IL-10 in our cohort is consistent with the clinical observation that MC generally does not feature prominent systemic inflammation [44]. At the same time, the relatively small sample size, particularly in the LC subgroup, cannot be excluded as a potential influence on these findings. Taken together, these results underscore the need for further research to determine whether the immunoregulatory environment in MC is confined to the intestinal mucosa or whether subtle systemic changes may also occur that remain undetected in small cohorts. Larger studies incorporating both tissue and plasma analyses will be essential to better define the role of IL-10 in the immunopathogenesis of MC and its potential to distinguish MC from other chronic inflammatory bowel diseases.

LPSs are glycolipid endotoxins originating from Gram-negative gut bacteria that may trigger an inflammatory response upon entering the systemic circulation and may also contribute to driving intestinal inflammation [27,45]. Under normal circumstances, an intact intestinal barrier prevents LPSs from entering the bloodstream; however, disruptions in barrier integrity—such as those occurring in chronic inflammatory intestinal diseases—permit LPS translocation into systemic circulation [27]. Several studies have reported elevated levels of LPSs and LPS-binding protein in both CD and UC [28,29,30]. In contrast, another study found that LPS levels in patients with UC and CD were not significantly increased compared with controls [31]. Nevertheless, evidence of prior LPS exposure was noted, primarily through elevated LPS-binding protein, with the authors suggesting that chronic exposure in IBD may lead to upregulated LPS clearance. This results in increased hepatic clearance of LPS-LPS-binding protein complexes and enhanced LPS uptake by liver macrophages. Similarly, in our study, LPS concentrations in UC patients were not significantly higher than in controls. To date, circulating LPS levels have not been systematically investigated in MC. However, several lines of evidence indicate that compromised intestinal barrier integrity is a central feature in MC pathogenesis. Increased mucosal permeability and inflammation in MC have been shown to improve following faecal stream diversion [32], while patients with CC exhibit increased mucosal bacterial uptake that persists even after successful clinical treatment [33]. Similarly, LC has been associated with impaired intestinal barrier function, as evidenced by downregulation and redistribution of tight junction proteins [34]. In this context, our study provides novel insight by evaluating plasma LPS levels in MC patients compared with healthy controls. Although no statistically significant differences were observed overall, CC patients demonstrated numerically higher LPS levels than controls, which may hint at the potential role of microbiome-derived signatures in CC.

This study has several limitations inherent to its exploratory design. The relatively modest sample sizes, particularly for lymphocytic colitis (LC; n = 16), limit statistical power for detecting small-to-moderate effect sizes, especially when comparing MC subtypes to healthy controls or ulcerative colitis (UC) groups. Post hoc power calculations (assuming two-sided α = 0.05 and observed means/standard deviations) indicated adequate power (~60–70%) for the significant FABP2 differences between collagenous colitis (CC) and UC, but substantially lower power (30%) for several non-significant comparisons, including FABP2 and LPS versus controls, and most LC analyses. This underpowering may have contributed to type II errors and underscores the preliminary nature of our findings. Furthermore, as a single-center study conducted at the Lithuanian University of Health Sciences, the cohort may not fully represent the broader spectrum of microscopic colitis patients across different populations, geographic regions, or healthcare settings. This limits the generalizability of our findings on circulating FABP2, IL-10, and LPSs as potential biomarkers. The cross-sectional design also precludes assessment of longitudinal changes in biomarker levels in relation to disease activity, treatment response (e.g., to budesonide), or clinical remission, which would be particularly valuable for understanding the dynamic role of intestinal barrier markers and immunoregulatory cytokines in MC pathogenesis. Finally, although patients were carefully selected according to established diagnostic criteria, we cannot exclude the potential influence of unmeasured confounders such as comorbidities (e.g., celiac disease, autoimmune disorders, or metabolic conditions) and concomitant medications (e.g., NSAIDs, PPIs, or other agents known to affect gut permeability or systemic inflammation). These factors could have modulated plasma biomarker concentrations independently of MC or UC status. Future multicentre, prospective studies with larger cohorts, detailed comorbidity profiling, medication reconciliation, and serial sampling will be essential to validate and extend these exploratory observations.

## 5. Conclusions

In this exploratory study, we observed significantly lower IL-10 levels in patients with active UC compared with both healthy controls and those with CC. Plasma FABP2 concentrations were higher in CC than in both active and inactive UC. However, both IL-10 and FABP2 demonstrated only low to moderate diagnostic accuracy, indicating limited potential as standalone biomarkers. Plasma LPS levels were numerically higher in CC compared with controls, though the difference did not reach statistical significance in all comparisons. None of the evaluated biomarkers reliably differentiated LC from controls or UC, an outcome that may be attributable to the relatively small number of LC cases included in the cohort. These preliminary findings highlight potential differences in circulating biomarker profiles between CC and UC but underscore the need for validation in larger, independent studies before considering any clinical application.

## Figures and Tables

**Table 1:** Demographic and clinical characteristics of the study population.

Variable	Controls	Active UC	Inactive UC	CC	LC
Number of subjects	52	43	43	45	16
Female, n (%)	27 (52)	31 (72)	23 (53)	38 (84)	12 (75)
Average age (years), mean (SD)	59 (4.9)	58 (8.8)	54 (12.4)	58 (13.4)	55 (16.1)
Average stools/day, mean (SD)	-	-	-	7.5 (3.2)	4.8 (2.5)
Average watery stools/day, mean (SD)	-	-	-	7.4 (3.3)	4.1 (2.7)
Average Mayo score, mean (SD)	-	7.8 (1.8)	1.7 (0.6)	-	-
Average Mayo endoscopic score, mean (SD)	-	2 (0.6)	0 (0.3)	-	-
Proctitis, n (%)	-	9 (21)	5 (12)	-	-
Left-sided colitis, n (%)	-	22 (51)	21 (49)	-	-
Extensive colitis, n (%)	-	12 (28)	17 (39)	-	-

UC: ulcerative colitis; CC: collagenous colitis; LC: lymphocytic colitis. For inactive UC, the greatest disease extent observed at the time of disease flare is reported.

## Data Availability

The data presented in this study is available on request from the corresponding authors. The data is not publicly available due to coded patient information present in the datasets and the restrictions of the ethical review board for public availability.

## Abbreviations

The following abbreviations are used in this manuscript:

MC	Microscopic colitis
CC	Collagenous colitis
LC	Lymphocytic colitis
FABP2	Fatty acid-binding protein 2
IL-10	Interleukin-10
LPSs	Lipopolysaccharides
IBDs	Inflammatory bowel diseases
UC	Ulcerative colitis
CD	Crohn’s disease
ROC	Receiver operating characteristic
CI	Confidence interval
AUC	Area under the curve
